# Nursing at Queen Charlotte's Lying-In Hospital

**Published:** 1887-02-05

**Authors:** Thomas Ryan

**Affiliations:** Secretary


### A Noble Profession: Nursing the Sick.

### NURSING AT QUEEN CHARLOTTE'S LYING-IN HOSPITAL.

By Thomas Ryan, Secretary.

I think it will be admitted generally that, while there is no calling for which educated women are more happily and especially fitted than that of nurse, it is not less true that there is no branch of nursing which is so absolutely and peculiarly their own, or which makes such exceptional demands upon their delicacy and refinement, as that of monthly nursing. This fact has of late years not escaped recognition, and it has been well said that, while a few years ago the number of women of the upper and middle classes who were to be found in the ranks of monthly nurses could be counted by units, they may now be reckoned by scores.

It will, I am sure, surprise many to learn that during the last ten years alone more than eleven hundred nurses have passed through a course of training in this hospital, and that last year the number trained was no less than one hundred and forty. A large proportion of these had already been trained in medical and surgical nursing at one or other of the metropolitan or provincial general hospitals; for instance, nurses have been received from the following London hospitals, the London, Guy's, St. Thomas's, St. Bartholomew's, St. George's, King's College, University College, Middlesex, and also from the Victoria Hospital for Children, and other special hospitals. The chief provincial institutions from which pupils have come, are the Manchester Royal Infirmary, the Liverpool Southern Hospital, the Norfolk and Norwich, the Royal Berkshire, the Devon and Exeter, the Nottingham General, and Addenbrookes at Cambridge; also the Sarah Acland Home at Oxford and several other nursing associations.

Before proceeding to describe our system of training, it is desirable that I should explain that the course of study necessary for a monthly nurse is much shorter than that which is considered essential for those undergoing training in medical and surgical nursing at a general hospital. The minimum term for which a nurse is received here is eight weeks, but they are encouraged to enter for twelve weeks, and many do so. Candidates may not be under twenty-one, nor exceed forty years of age, they must produce testimonials of moral character and a medical certificate of health, and, of course, be able to read and write.

Pupils are received weekly, the day of entry being Monday; and to ensure commencing on any given date, application should be made about three months beforehand. The fees are ten guineas and a half for eight weeks, or fifteen guineas for twelve weeks. It is not absolutely necessary that the dresses worn should be of a uniform description, but they must be of a washing material, and either white or a very light print. Caps and aprons of the same pattern as those in use generally in the London hospitals must be worn.

The wards throughout the hospital contain three beds each, two for patients and one for the nurse so that each nurse has charge of two patients with their infants, and tends them day and night, in the same way as they would in private nursing, except that there is a night sister, with four nurses under her, to exercise general observation over all the patients, to attend to those admitted during the night, and to prepare such nourishment as is needed. The nurses on the average have the custody of three sets of cases—that is six patients—during their eight weeks course. They receive their practical training from the matron and sisters, and come under medical observation twice daily, as the patients are visited every morning by the Resident Medical Officer and each afternoon by the Visiting Physician. This practical training is supplemented by lectures which are delivered twice weekly by the Visiting Physicians. These lectures are a recent extension of the system of training and it is of course superfluous to say that they have raised it to an incomparably higher level, and immensely enhanced its value. It is indeed difficult to over-estimate the importance of these lectures to the nurses, or to speak too greatly of the kindness and patience of the Visiting Physicians in giving them.

The comfort of the nurses has been amply and carefully provided for, and opportunity was taken, when the new wing was built last year, to make several improvements in this particular. Extra bed rooms were provided on the top floor for their use when off duty, and a well-appointed lavatory, and a bath-room for their sole use, both with hot and cold water, were fitted up on the same floor. A commodious, light, and airy dining room was also provided in the basement of the new wing, in place of the old room which was not quite suitable for the purpose. There is a small chapel in the hospital where services are held by the Chaplain on all Sundays and Holy Days throughout the year. All who desire may attend, but there is no pressure, direct or indirect, put upon any nurse, and facilities are afforded to those who may not be members of the Church of England to attend a place of worship, if they wish to do so, every other Sunday.

I have omitted to state that at the completion of her course of training, each nurse, if found qualified, is granted a parchment certificate, testifying to her competence to discharge efficiently the duties of monthly nurse, under the hands of the visiting physicians and the matron, and countersigned by the secretary.

I may be permitted briefly to point out that, as this institution is now by far the largest lying-in hospital in Great Britain, and as the number of its in-patients exceeds that of all the other metropolitan lying-in hospitals combined, it offers exceptional and unrivalled advantages to those desirous of being thoroughly trained either in midwifery or monthly nursing.

A word as to the prospects of monthly nurses holding our certificate may not unfittingly close these remarks. Probably no hospital has received so large and unbroken a share of Royal patronage and support. From Her Majesty Queen Charlotte, after whom it is named, to Her Majesty Queen Victoria, every sovereign, and nearly every member of the Royal Family has contributed generously to its funds, and it owes its very existence to His Royal Highness the late Duke of Sussex. On this account, and by reason of the extent of its work, it is well known throughout the kingdom, and its nurses have been very successful in obtaining employment in families of the highest position not even excepting the Royal Family itself: and I have every reason to believe that our nurses have uniformly experienced very little difficulty, after the first start, in obtaining regular and remunerative employment.

